# Effect of graphic warning label on acute changes in waterpipe tobacco smoking behavior, biomarkers of exposure and harm, and subjective effects in a randomized trial

**DOI:** 10.1016/j.addbeh.2025.108520

**Published:** 2025-10-21

**Authors:** Marielle C. Brinkman, Toral Mehta, Michael L. Pennell, David Angeles, Soliana Kahassai, Chieh-Ming Wu, Hayley Curran, Brittney Keller-Hamilton, Elizabeth G. Klein, Megan E. Roberts, Paul Nini, Olorunfemi Adetona, Joanne G. Patterson, Darren Mays, Lucia Mandarano, Emma Jankowski, Annabelle Thomas, Amy K. Ferketich

**Affiliations:** a Division of Epidemiology, College of Public Health, The Ohio State University, College of Public Health, United States; b Center for Tobacco Research, The Ohio State University Comprehensive Cancer Center, United States; c Division of Biostatistics, College of Public Health, The Ohio State University, United States; d Division of Environmental Health Sciences, College of Public Health, The Ohio State University, United States; e Divison of Medical Oncology, College of Medicine, The Ohio State University, United States; f Division of Health Behavior and Health Promotion, College of Public Health, The Ohio State University, United States; g Department of Design, College of Arts and Sciences, The Ohio State University, United States

## Abstract

**Introduction:**

Waterpipe (WP) smoking is commonly misperceived as less harmful than cigarette smoking. Graphic warning labels (GWLs) may correct misperceptions and reduce WP smoking. We determined the impact of a GWL placed on a WP on short-term smoking behaviors and other outcomes among young adults who smoke WP.

**Methods:**

Young adults, ages 21–35 years, who smoke WP were randomized to a control (Visit1 = Blank, Visit2 = Blank label) or experimental (Visit1 = Blank, Visit2 = GWL, Visit3 = GWL) group. Participants smoked a research-grade WP in the lab *ad lib* to satiation, for a maximum of 60 min, for up to 3 visits, each separated by a week. Puffing topography was measured continuously throughout the session. Exhaled CO, harm perceptions, and subjective effects were measured before and after each smoking session. Outcome data were analyzed using linear mixed models to account for incomplete, repeated measurements.

**Results:**

There were no significant differences in study outcomes within and between assigned groups, except for the following from Visit1 and Visit3 in the GWL group: 1) a reduction in puff volume (p = 0.048); 2) a reduction in good taste and calmness (p = 0.027 and 0.007, respectively); and 3) a reduction in being confused after smoking (p = 0.042).

**Conclusions:**

GWLs on WPs may not be the sole effective tool for reducing the harm from WP smoking. More research is needed to determine if WP interventions aimed at improving population health should include a wider variety of tools, such as education, cessation services, and product standards that limit the appealing aspects of WP tobacco.

## 1. Introduction

Adolescents and young adults are susceptible to waterpipe (WP) smoking. (Orlan et al., 2019; Alalwan et al., 2024) The proximity of WP cafés to college campuses and availability of flavorful tobacco that masks harshness and enhances taste contributed to the dramatic increase in WP smoking prevalence in the United States (U.S.) for about a decade, starting in the early 2000′s. (Maziak et al., 2004; Kates et al., 2015; Maziak et al., 2004; Rastam et al., 2004) While the prevalence of past 12-month WP smoking has decreased considerably since 2014 among U.S. adolescents and young adults, it was still 3 % among high school seniors and 8 % among young adults aged 19–30 years in 2022. (Patrick et al., 2023; Miech et al., 2023).

WP smoking is commonly perceived as less harmful than cigarette smoking. (Villanti et al., 2015; Gathuru et al., 2015; Akl et al., 2013) Yet, WP tobacco smoke contains addictive levels of nicotine (Aboaziza and Eissenberg, 2015; Kulak et al., 2017) and concentrations of harmful and potentially harmful constituents that are 10–100 times higher than those in cigarette smoke. (Shihadeh et al., 2014) In fact, adolescent WP users may develop nicotine dependence symptoms earlier than adolescent cigarette users. (Ebrahimi Kalan et al., 2020) Adverse health effects associated with WP smoking among adolescents and young adults include acute lung infection and injury, lung function decline, carbon monoxide (CO) poisoning, oral and systemic genotoxicity, psychological and neurological effects, and asthma. (Adetona et al., 2021).

To correct misperceptions about the health risks of WP smoking, effective health communication strategies need to be developed and evaluated. One potentially effective communication is a health warning label, which has successfully increased public knowledge of the harmful effects of cigarettes, quit intentions, and calls to quit lines, as well as decreasing smoking prevalence. (Hammond et al., 2007; Hammond, 2011; Monarrez-Espino et al., 2014; Noar et al., 2016) The U.S. Food and Drug Administration (FDA) has the authority to mandate health warning labels on WP products, such as tobacco packages and WPs. (Food and Drug Administration, 2016) Currently, WP tobacco packaging must include the following text-only message: “WARNING: This product contains nicotine. Nicotine is an addictive chemical.” This is the only federally mandated warning label associated with WP smoking in the U. S., as warning labels on WP components or parts are not required. (FDA) While this regulation informs WP smokers who buy WP tobacco in commercial packaging, it may not be effective for the majority of WP smokers for three important reasons. First, many U.S. young adults smoke WP in cafés where the WP is brought to them, so they are unlikely to encounter WP tobacco packaging. (Salloum et al., 2016) Second, the cigarette literature indicates that graphic, not text-only, warning labels are more effective. (Hammond, 2011) Third, the message about nicotine is less effective at promoting thoughts about cessation among WP smokers than other messages. (Islam et al., 2016; Sutfin et al., 2021).

While there is limited research on WP warning labels compared to cigarettes, findings suggest that messages emphasizing the health and safety of children (Islam et al., 2016) or comparing WP smoking to smoking 100 cigarettes (Sutfin et al., 2021) would be effective. Additionally, the mouthpiece, stem, or hose were reported to be the best locations for WP warning labels. (Islam et al., 2016) Less is known about the potential for health warning labels to impact acute WP smoking behaviors. Two non-randomized crossover trials (one pilot-level) reported the results from laboratory-based studies that examined the change in smoking behavior from a no-health warning label condition to a graphic warning label (GWL) condition. (Maziak et al., 2019; Gautam et al., 2023) In the pilot study, smoking a WP with a GWL resulted in lower exhaled CO boost, fewer puffs per smoking session, and a less positive smoking experience compared to smoking a WP with no label. (Maziak et al., 2019) A recent report of this pilot study’s expansion to 100 WP smokers shows the GWL condition resulted in minor but significant decreases in smoking behavior (12 % fewer puffs, smoked for 2 % less time, puffed for 3 % less time) and a significant decrease in exhaled CO boost (22 % less). (Shaukat et al., 2025) The second study indicated participants’ typical WP smoking frequency was related to acute smoking changes. Participants who smoked WP less frequently (< once per week) reduced their WP smoking time to a greater extent and took fewer puffs when smoking a WP with a GWL compared to those who smoked WP more frequently (≥ once per week). (Gautam et al., 2023).

This study sought to address the data gap surrounding the efficacy of GWLs to effect short-term smoking behavior by incorporating a parallel arm control group and randomized design. Building on our previous work developing a GWL (Moumen et al., 2020) and examining its optimal location on a WP, (Klein et al., 2021) we conducted a two-arm, parallel randomized trial (1:1 allocation ratio) to compare two visits (V1, V2) with a blank label only condition (Blank-Blank) to a blank label followed by two more visits with a GWL condition (Blank-GWL-GWL). In this controlled laboratory setting, the difference between arms is the GWL, and thus any changes measured in smoking behavior, toxicant exposure, and subjective effects can be more strongly attributed to the presence of the GWL. The design also protects against testing effects, or systematic changes in participant behavior that can occur because of participants completing the WP smoking task more than once. The objective of this study was to determine the impact of a GWL placed on a WP on short-term smoking behaviors, a biomarker of exposure, subjective effects, and harm perceptions among young adults who smoke WP. We hypothesized that exposure to a health warning label would result in puffing less frequently for a smaller total volume puffed and result in greater perceptions of harm.

## 2. Methods

### 2.1. Participants

Participant enrollment and study procedures occurred between July 2021 and June 2022, in Columbus, Ohio. Using social media, print advertisements, flyers, and referrals, we recruited a convenience sample (n = 92) of people who smoke WP (3 or more times in past 30 days), between ages 21–35 years, and screened for eligibility by telephone. The smoking rate was chosen to satisfy ethical considerations, given that enrolled participants would be asked to smoke WP up to three times in three weeks. Exclusion criteria included current or planned pregnancy, currently breastfeeding, evident intoxication at any clinic visit, active respiratory infection, and history of significant smoking-related disease. To simulate potential policy actions, participants were not fully informed of the primary objective of the study, which was to evaluate the effect of a GWL, but were instead told that the goal of the study was to evaluate WP puffing behaviors and any lung or cellular damage from WP smoking.

### 2.2. Study protocol

#### Overview:

Participants were randomized to a Control or Experimental. The Control group attended two laboratory visits (V1 = Blank, V2 = Blank condition) and the Experimental group attended three visits (V1 = Blank, V2 = GWL, V3 = GWL condition). All smoking sessions were separated by one week and took place in a laboratory smoking room that had sufficient ventilation (>12 ACH) so that room air CO never exceeded 35 ppm. Each session took place within one hour of the same time of day after ≥ 8 h of combustible tobacco smoking abstinence (exhaled CO < 10 ppm). (Cobb et al., 2015) Participants who were assigned to the Control group received $90 for completing both visits and those assigned to the Experimental group received $180 for completing three visits.

#### Laboratory WP Smoking Procedures:

At V1, participants provided their signed, informed consent, answered further questions about eligibility, completed a pregnancy test (if applicable), had their weight and height measured, and were randomized (Block) to either the Control or Experimental group.

At each visit, participants completed an electronic survey before and following the WP smoking protocol. At V1, the survey included questions about demographics, childhood and current socioeconomic status, tobacco use history (WP and other tobacco products), WP smoking behaviors (e.g., at home, at a café), as well as two nicotine dependence scales: the Lebanese WP smoking dependence scale (LWDS-11) (Salameh et al., 2008) and the Hooked on Nicotine Checklist (HONC). (DiFranza et al., 2002) At all visits, the pre-visit survey included the following: 1) the Direct Effects of Nicotine scale (DENS); (Evans et al., 2006) 2) the Minnesota Withdrawal Scale modified for WP (MWS); (Hughes and Hatsukami, 1986) 3) relative (to cigarettes and e-cigarettes) WP harm perceptions; 4) absolute WP harm perceptions; and 5) the questionnaire on smoking urges (QSU) adapted for WP. (Tiffany and Drobes, 1991) The post-visit survey included these same scales plus the Direct Effects of Tobacco scale (DETS). (Foulds et al., 1992).

#### WP Smoking Protocol:

For greater precision and accuracy, a validated and user-accepted research grade waterpipe (RWP), that records user puffing topography data was used. (Brinkman et al., 2016; Kim et al., 2016; Brinkman et al., 2020) Study personnel prepared the RWP and participants smoked *ad lib* to satiation, for a maximum of 60 min (no minimum smoking time was required). The RWP head was filled with 15 g tobacco in the participant’s preferred flavor (Double Apple, Mint, Gum Mint, Lemon Mint, Orange, and Rose flavors; all Nakhla, Egypt). The tobacco flavor each participant selected was smoked for all visits. Puffing topography was measured continuously throughout the session by the RWP. Exhaled CO was measured using a handheld electro-chemical cell (CoVita Smokerlyzer, Santa Barbara, CA) before the smoking session and after 45 min of ad lib smoking (if the participant chose to smoke that long). If, after smoking for 45 min, a participant’s exhaled breath CO was greater than 50 ppm, the smoking session was discontinued and a final exhaled breath CO was collected. If not, the participant was allowed to continue smoking as they desired, for a maximum of 60 total minutes. Immediately following the smoking session, a final exhaled CO level was measured. For more details, see Supplemental Information.

#### Intervention:

**Fig. 1.**
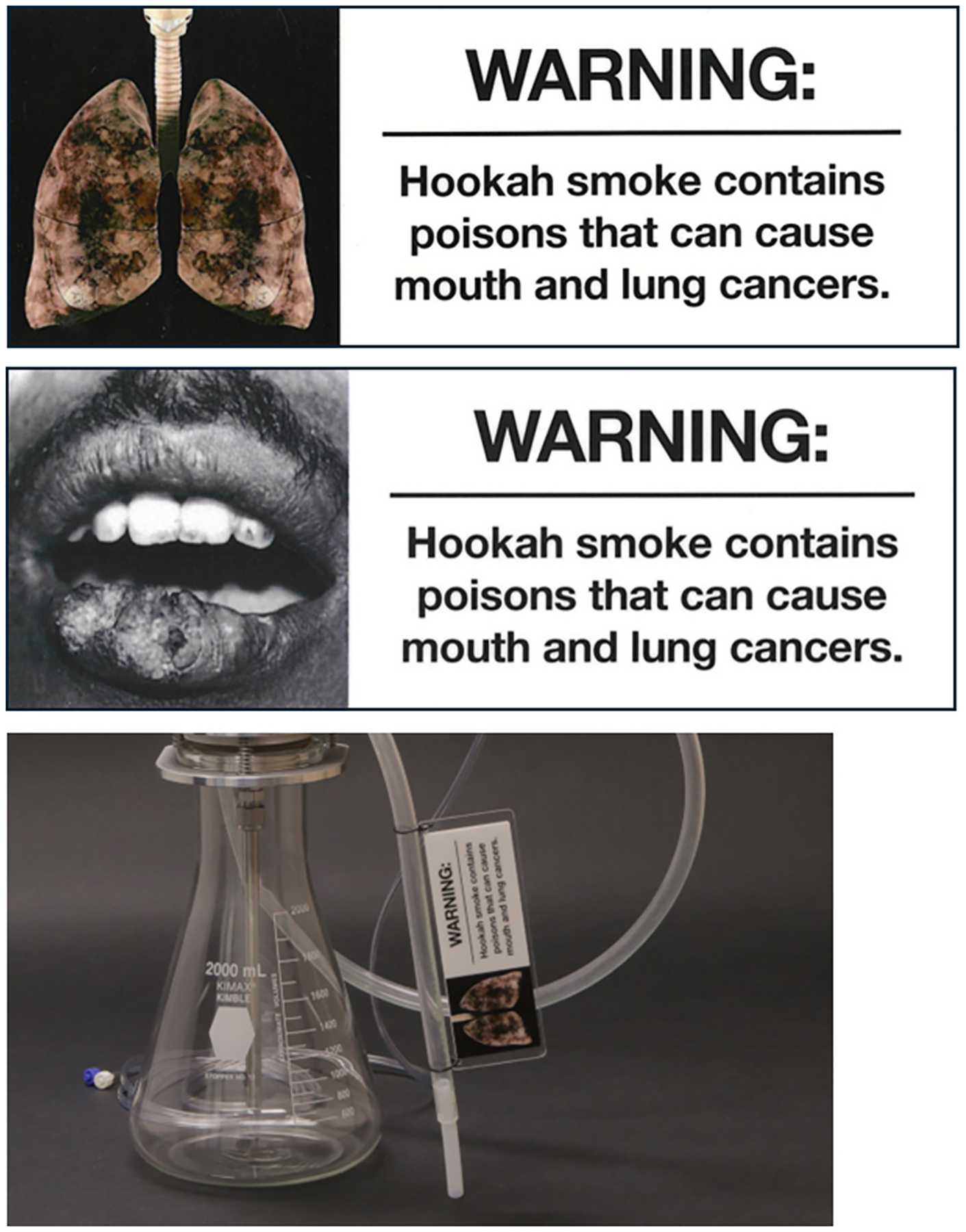
The graphic warning label had the same message on both sides of it, but two different pictures (top and middle), and was placed on the hose, just below the mouthpiece of the research-grade waterpipe (bottom). The blank label, which contained no text nor images, was identical in size and placement (not pictured).

The GWL message was developed and evaluated by our team. (Moumen et al., 2020; Klein et al., 2021) Briefly, we tested the FDA mandated warning message (*WARNING: This product contains nicotine: Nicotine is an addictive chemical*.) and several additional messages that have been used in other countries or on other tobacco product packaging among young adults. (Moumen et al., 2020) The message that had the highest rating of effectiveness and lowest level of reactance was *WARNING: Hookah smoke contains poisons that cause lung and oral cancers*. This message was paired with graphics that depicted lung and oral cancers (Fig. 1). Eye tracking indicates that, regardless of location on the WP, the presence of a GWL attracts visual attention. (Klein et al., 2021) We selected the hose just below the mouthpiece so that the GWL will be seen even if the WP is on the floor and/or in dim lighting. Thus, both conditions included an identically sized label (GWL contained text and graphics, blank label contained no text nor graphics) placed in the same location.

### 2.3. Waterpipe Configuration and smoking topography data collection

Details regarding the setup, precision, accuracy and acceptance of the RWP are described elsewhere; (Kim et al., 2016; Brinkman et al., 2020; Brinkman et al., 2016) see Supplemental Information.

### 2.4. Sample size and measures

The sample size for each group (Control and Experimental) was 46 participants; see Supplemental Information. The primary dependent variables were acute smoking-related measures: 1) puffing topography [puff volume (L), puff duration (s), average puff flow (L/min), total puff volume (L), total number of puffs, total puffing time (min), and total time smoking (min)]; 2) consumption (total tobacco consumed (g), total charcoal consumed (%)); and 3) exhaled CO boost (ppm) from pre- to post-smoking.

Secondary dependent variables included the DENS, (Evans et al., 2006) the DETS, (Foulds et al., 1992) MWS, (Hughes and Hatsukami, 1986) the Brief WP Smoking Urges Questionnaire, (Tiffany and Drobes, 1991) and absolute harm perceptions; see Supplemental Information.

The primary independent variable was the condition: Blank label (Control) vs. GWL (Experimental).

### 2.5. Data analysis

Analyses adhered to intention-to-treat principles. Linear mixed models were used to model smoking behavior across visits, which accounted for repeated measurements on individual participants and missed visits. Of primary interest was the difference in the change in outcomes from V1 to V2 by group, which was determined by condition-by-visit interactions in our models. Intra-group comparisons of the changes over time were performed by stratifying our mixed models by group and performing multiple comparisons of the means at each visit. Holm’s method was used to control overall type-I error rate across these comparisons. Further details are provided in the Supplemental Information. We examined the difference (Experimental – Control) in change between visits 1 and 2, adjusting for race (NH white, NH black, other), age, sex, years of education, use of cigarettes, cigars, and e-cigarettes (ever/never), and hookah use (last 30 days, lifetime use at private residence, and lifetime use at café; each coded as five times or less vs. more than 5 times).

## 3. Results

### 3.1. Participants

A total of 92 participants (n = 46 per group) enrolled in the study and were included in the analyzed sample; 12 participants did not complete all visits (11 % lost to follow-up in Control, and 15 % in Experimental). Participants had a mean age of 26.2 ± 4.0 years, were roughly half female (51 %), almost half Black (43 %) and mainly non-Hispanic or Latino (95 %). Participant demographics and WP dependence and WP use did not differ by group, as shown in Table 1.

### 3.2. Smoking Outcome results

Across all groups and visits, average tobacco consumption ranged from 2.57 to 2.83 g/visit and average charcoal consumption ranged from 66.6 % to 69.8 % of the initial mass of the charcoal (Table 2). Average CO boost ranged from 46.5 to 55.1 ppm. Average puffing topography results were the following: 1) puff volume ranged from 0.70 to 1.15 L; 2) puff duration ranged from 2.6 to 3.5 s; 3) average puff flow ranged from 11.5 to 17.4 L/min; 4) total puff volume ranged from 44.8 to 64.7 L; 5) total puffs ranged from 54.2 to 72.5; 6) total puffing time ranged from 2.7 to 3.2 min; and 7) total smoking time ranged from 36.3 to 39.0 min. There were no significant differences in these measures within and between assigned groups, except a significant decrease in puff volume, 20 % reduction (p = 0.048), from V1 to V3 in the GWL group. There were no significant differences within and between groups when dropouts were excluded; see Supplemental Table ST-1.

### 3.3. Other outcomes

The average DETS items (Table 3) suggest that positive ratings, such as satisfying, pleasant, good taste, and calmness, resulted in averages above 30 (on a 0–100 scale) for most visits. Negative ratings, such as confusion, dizziness, and sick, had averages under 20. There were no significant differences in these measures within and between groups except for a significant decrease in good taste (18 % reduction) and calmness ratings (43 % reduction) in V3 compared to V1 for the GWL group (p = 0.027 and 0.007, respectively).

Most of the DENS items increased following the WP smoking session with and between groups, except for nervous and sweaty, which increased after some sessions in both groups (see Supplemental Table ST-2). The pre- to post-smoking session changes in any of the DEN items were not significantly different within and between groups except for a much smaller decrease in being confused, (p = 0.042), after smoking in V1 compared to V3 in the GWL group.

Most withdrawal symptoms (Table 4) decreased following the WP smoking session (except for restlessness, hunger, drowsiness, and sweet desire). Finally, smoking urges (Table ST-3) decreased following the smoking sessions. However, differences between and within groups were not statistically significant for these measures.

Differences in harm perceptions were not statistically different between and within groups (see Supplemental Table ST-4).

## 4. Discussion

This is the first randomized, controlled laboratory smoking study to show that a GWL located on the WP hose near the mouthpiece is not effective in reducing acute toxicant exposure and positive experiences from WP smoking for young adults who smoke WP. Although recent evidence shows that GWLs, instead of text-only warning labels, elicit greater attention among people who smoke WP, (Gautam et al., 2023) we observed no changes in puffing behavior, exhaled CO boost, subjective smoking experiences, and absolute and overall harm perceptions associated with smoking a WP for two sessions, each session separated by one week.

The results of our study conflict with a controlled laboratory pilot (n = 30) study that was a non-randomized repeated measures crossover study, conducted by Maziak et al. (Maziak et al., 2019) They linked the presence of a GWL on the water bowl to reductions in WP smokers’ positive smoking experiences, puffing behavior, exhaled CO boost, and increases in the appreciation of the harm from WP smoking. The GWLs used in both studies included scientifically credible statements about cancer as a risk factor associated with WP smoking and included pictures of diseased mouth and lungs. Participants in our study were older (four years on average) had smoked WP ≥ 3 times in the past month, and thus may have been more nicotine dependent. The Maziak et al. study included less frequent WP smoking (1–3 WPs in the past 30 days), and a cigarette smoking frequency of ≤ 5 cigarettes in the past 30 days. (Maziak et al., 2019) One fourth of our sample smoked cigarettes in the past 30 days, with 61 % smoking more than 5 cigarettes during that time (exceeding the study exclusion criterion in Maziak et al.). Our participants’ average HONC and LWDS-11 indicated moderate to high nicotine dependence. Maziak et al., recently reported a larger study (n = 60), where WP smoking frequency was examined as a proxy for nicotine dependence. (Gautam et al., 2023) There, a GWL on the WP bowl only lowered satisfaction regarding the smoking experience in people who smoked WP at a high frequency (≥1 time/week); reductions in smoking time and number of puffs were found in low frequency (<1 time/week) WP smokers who were potentially less dependent on WP smoking. This result, in combination with our findings, indicate that the loss of autonomy associated with nicotine dependence may lower the efficacy of a GWL to reduce acute WP smoking behavioral changes and increase harm perceptions. However, our ad hoc analysis of the correlation between measured participant smoking outcomes and nicotine dependence score (LWDS-11) indicate the strength of association is strongest for tobacco consumption and CO boost, but still somewhat weak (r < 0.4), as shown in Table ST-5.

Given that tobacco flavor was only standardized across a participant’s visit once they selected it at Visit 1, and not across groups, we examined whether puffing behavior varied by flavor. There were minor differences in flavor selected across the two groups (see Table ST-6). Although tobacco consumption did vary by flavor, with all other flavors consumed ~ 70 % less than Double Apple, this did not translate to differences in puffing behavior or CO exposure by flavor (see Table ST-7). Thus the consumption difference is more likely due to the additives in the Double Apple tobacco rather than participant liking. Therefore, we did not adjust for flavor as a covariate in our analyses.

Our study was designed to simulate the WP café experience of seeing WP GWLs for the first time on a prepared WP. It may be that two ~ 30-minute exposures to a single GWL, separated by one week, are not enough to have a significant effect on nicotine dependent WP users’ smoking behaviors. The GWLs on cigarette packages are seen much more frequently than this because users must interact with the package throughout the day and at a minimum of 20 times to smoke the cigarettes inside of it. In addition, GWLs on cigarette packaging may be effective in dissuading non-smoking individuals from trying cigarettes, because these packages are more commonly seen by non-users when the product is being used, or left in the viewable environment. Repeated exposure to the GWL may be critical for influencing harm perception in WP smokers, as indicated by a previous examination of PATH data which showed that WP smokers exposed to GWLs on WP tobacco packaging in Wave 1 were more likely to perceive WP as more harmful than cigarette smoking at Wave 2. (King et al., 2019) Our study design does not determine whether a GWL on a hookah is effective in reducing hookah initiation because repeating this study design in a cohort of young adults who do not smoke hookah would be unethical. Previous work has shown that when exposed to WP GWLs, nonsmokers score higher in harm perception metrics than established WP smokers, (Mostafa et al., 2021) and thus GWLs on WPs may be more effective as deterring initiation than influencing established WP smokers to quit. Novel approaches are needed to understand how GWLs on WPs in WP cafés may impact a non-smoking person’s decision to initiate WP smoking for the first time.

Our study design corrects for testing effects, or systematic changes in participant behavior that can occur when participants complete a task multiple times. Our analysis shows that, although participants knew they could leave the laboratory soon after they had completed their WP smoking session, their average smoking time and puffing behavior largely did not change over time. This lends additional support to our hypothesis that nicotine dependence was the primary driver of our participants’ smoking behaviors.

Although our participants were interacting with the hose near the mouthpiece with every puff they took, the label itself was not necessarily directly in their line of vision when they were drawing smoke through the WP. The hose-near-the-mouthpiece location was chosen in agreement with previously surveyed experts, (Mostafa and Mohammed, 2017) and as a strategy to overcome the low lighting and placement of the WP on the floor, conditions that are typical of WP cafés in the Columbus, Ohio, area. Our results suggest that to be effective, two GWL exposures may not be enough. Ways to make GWLs for hookah more likely to be seen include covering more than half the WP, and placement on the WP tobacco menu, tabletops, and café walls where WP is being served. Regulatory requirements in Turkey, including that more than 65 % of the WP must be covered by the GWL, and posted GWLs in smoking areas, are associated with lowered WP smoking. (Erdol et al., 2015).

### 4.1. Strengths and Limitations

The main strength of this study is the rigorous design. By randomizing participants to group, and having each group start with a blank label, we were able to compare participants between groups (Blank Label vs. GWL) but also within each group to determine the changes from V1 to V2 (and, further, V2 to V3 in the GWL group). Each group performed a test and re-test of the condition they were randomized to, and results showed their puffing behavior was stable over the 2- or 3-week period. Another strength was our scientific approach to: 1) develop the optimal GWL message and image using qualitative research methods (Moumen et al., 2020) and 2) determine where best to place it using an eye-tracking study. (Klein et al., 2021) A final strength is the diversity of participants in the sample, as nearly half identified as Black or two or more races. Recent 2023 nationally representative data for ever use of hookah show slightly higher rates of trial among non-Hispanic Black middle and high school students. (Birdsey et al., 2023) The sample also had diversity with respect to childhood and current socioeconomic status.

As with all laboratory smoking studies, a limitation is that the waterpipe and smoking environment differ from a natural setting, which may impact smoking behaviors. For this study, the laboratory setting may not reflect one’s usual WP smoking patterns because they were in the smoking room alone (vs. smoking in a social setting). Further, given this convenience sample of WP smokers, the results are not generalizable to the U.S. population, nor to nicotine-naiive individuals that are considering trying WP smoking. Given our sample size, we were not able to examine effect moderation by nicotine dependence. In addition, the waterpipe used looks different from most commercial waterpipes, although it has identical components (hose, water bowl, mouthpiece, etc.). Participants were allowed to use their smartphones to watch videos, play games, listen to music, and text with friends. Both of these things could affect how much attention participants paid to the warning label. While phone use may have been “normal” for a WP smoking session for some of our participants, they might not have been “normal” for all participants (e.g., perhaps some participants usually talk with a friend while smoking at a café). Finally, this study had a limited exposure dose to the GWL. Moreover, the amount of time spent attending to the GWL was not measured. Future research would benefit from using eye-tracking or some other method to assess the amount of time people view the GWL while smoking. Because of these limitations, conclusions regarding the efficacy of GWLs placed on the WP should take this into consideration.

## 5. Conclusions

WP smoking is often the first combustible tobacco product tried by adolescents, (Villanti et al., 2015; Sharma et al., 2020) and there is longitudinal evidence supporting its role as a gateway to cigarette smoking. (Watkins et al., 2018) Accordingly, the U.S. and other nations share the same goal with respect to WP smoking: adopt meaningful regulatory strategies that address misperceptions about the harm of WP smoking and result in short- and long-term behavioral changes for a healthier population. There is now a moderate body of evidence indicating that, when educated about the harms via exposure to a GWL, young adult WP smokers increase their intentions to quit. (Jebai et al., 2023; Farran et al., 2021) However, based on our results, GWLs on the WP’s hose may not be the sole effective tool for influencing actual human smoking behavior in established WP smokers. Short-term puffing behavior and subsequent toxicant exposure appear to be largely governed by nicotine dependence, but monitoring smokers’ attention to the warning labels placed on WPs is key to a definitive understanding of this. More research is needed to determine if repeated exposure to WP graphic warning labels on the WP and tobacco packaging correlate to change in smoking behavior among established WP smokers. Although more costly than mandated graphic warning labels, a combined strategy of graphic warning labels and WP-specific, evidence-based education and cessation tools may work in tandem to dissuade both young adult non-smokers from initiating WP and help people who smoke WP to quit. WP interventions aimed at improving population health may need to rely on a wider variety of tools, such as large-scale public education, (Mostafa and Mohammed, 2017) accessible cessation services, (Erdol et al., 2015) and state or federally mandated product standards that limit the appealing aspects of WP tobacco. (Keller-Hamilton et al., 2023; Mays et al., 2021; Bhatnagar et al., 2019; Centers for Disease Control and Prevention, 2014).

## Supplementary Material

1

## Figures and Tables

**Table 1:** Demographic and tobacco use characteristics of participants, by condition.

	Control (n = 46) Blank Label	Experimental (n = 46) GWL^A^
Years of Age, mean (SD)^B^	26.3 (4.1)	26.2 (4.0)
Gender, number (percent)		
Male	21 (45.7)	24 (52.2)
Female	25 (54.4)	22 (47.8)
Race/ethnicity, number (percent)		
Non-Hispanic White	25 (54.3)	15 (32.6)
Non-Hispanic Black	14 (30.4)	24 (52.2)
Hispanic	4 (8.7)	3 (6.5)
Two or more races	1 (2.2)	0 (0.0)
Other	2 (4.4)	4 (8.7)
Childhood SES, number (percent)		
Barely enough to just enough	14 (30.4)	18 (39.1)
Solidly middle class	22 (47.8)	20 (43.5)
Above middle class	10 (21.7)	8 (17.4)
Current SES, number (percent)		
Working/lower-middle to middle	34 (75.6)	38 (82.6)
Upper-middle to upper class	11 (24.4)	8 (17.4)
Years of education, mean (SD)	15.7 (2.5)	14.6 (3.1)
Tobacco use history, number (percent)		
Ever cigarette smoker	31 (68.9)	23 (50.0)
Current cigarette smoker	11 (28.2)	12 (26.7)
Ever e-cigarette user	36 (78.3)	25 (54.4)
Current e-cigarette user	24 (60.0)	23 (51.1)
Ever cigar/cigarillo smoker	30 (66.7)	29 (63.0)
Current cigar/cigarillo smoker	10 (23.8)	13 (28.9)
Years of age when first tried waterpipe, mean (SD)^c^	18.9 (2.2)	18.6 (2.8)
Location where first tried waterpipe, number (percent)		
In a cafe or restaurant	26 (56.5)	25 (54.4)
In my own home	5 (10.9)	6 (13.0)
At a family member’s home	3 (6.5)	2 (4.4)
At a fraternity or sorority house	0	2 (4.4)
At a friend’s home	11 (23.9)	10 (21.7)
Other	1 (2.2)	2 (4.4)
Waterpipe smoking in past month, number (percent)		
1–5 times	19 (41.3)	22 (47.8)
6–10 times	8 (17.4)	5 (10.9)
11 or more times	19 (41.3)	19 (41.3)
Lifetime waterpipe smoking in cafe, number (percent)		
0–1 times	0 (0.0)	2 (4.4)
2–5 times	6 (13.0)	4 (8.7)
More than 5 times	40 (87.0)	40 (87.0)
Lifetime waterpipe smoking in home, number (percent)		
0–1 times	1 (2.2)	0 (0.0)
2–5 times	6 (13.0)	5 (10.9)
More than 5 times	39 (84.8)	41 (89.1)
HONC score, mean (SD)^C^	1.65 (1.91)	1.67 (2.22)
LWDS-11 score, mean (SD)^D^	8.05 (3.86)	8.67 (3.73)
Own their own hookah, number (percent)	34 (73.9)	31 (67.4)

A GWL = graphic warning label group.

B SD = standard deviation

C HONC = Hooked on Nicotine Checklist.

D LWDS-11 = Lebanese Waterpipe Dependence Scale.

**Table 2:** Smoking Outcome Data - Measures are Mean (Standard Deviation).

							Intra-Group Comparisons: p-value^A^				Inter-Group Comparison of V1:V2 Change	
Measure	Units	Control (C)		Experimental (E)			C	E			Adj Diff^B^	p-value
		V1 Blank	V2 Blank	V1 Blank	V2 GWL	V3 GWL	V1:V2	V1:V2	V2:V3	V1:V3		
No. Observ.		46	41	46	39	39						
**Consumption**												
Tobacco Cons	g	2.73 (1.32)	2.83 (1.54)	2.77 (1.28)	2.63 (1.04)	2.57 (1.12)	1.000	1.000	1.000	1.000	−0.35 (0.36)	0.337
Charcoal Cons	%	68.8 (10.3)^C^	69.8 (10.8)	68.1 (11.4)^C^	68.8 (11.8)^D^	66.6 (11.7)	0.692	0.692	0.195	0.557	0.69 (2.37)	0.773
**Biomarker of Combustion**												
CO Boost	ppm	49.5 (30.9)	46.5 (33.4)	55.1 (35.4)	47.6 (23.6)	47.4 (25.3)	1.000	0.497	1.000	0.711	−2.7 (6.5)	0.674
**Puffing Topography**												
Puff Volume	L	0.94 (0.89)	0.70 (0.46)	1.15 (1.01)	1.02 (0.91)	0.78 (0.63)	0.397	0.397	0.397	0.106	−0.11 (0.20)	0.583
Puff Duration	s	2.9 (1.6)	2.6 (1.4)	3.5 (1.9)	3.1 (1.7)	2.8 (1.4)	0.513	0.513	0.278	**0.048** ^E^	−0.1 (0.3)	0.722
Avg Puff Flow	L/min	17.4 (7.8)	16.0 (6.9)	17.6 (7.8)	17.5 (7.5)	15.5 (6.4)	0.851	0.851	0.851	0.646	−1.0 (1.5)	0.518
Total Puff Vol	L	64.7 (88.8)	51.0 (46.4)	62.3 (68.0)	61.6 (74.5)	44.8 (38.7)	0.759	0.922	0.509	0.411	−2.1 (16.3)	0.899
Total Puffs		67.8 (56.7)	72.5 (56.0)	54.2 (35.8)	60.6 (39.5)	57.2 (38.3)	0.423	0.320	0.812	0.812	−1.4 (6.2)	0.822
Total Puffing Time	min	3.2 (2.9)	3.1 (2.2)	3.1 (2.4)	3.2 (2.5)	2.7 (1.8)	1.000	1.000	0.674	0.674	−0.1 (0.4)	0.826
Total Smoke Time	min	38.2 (14.6)	39.0 (15.2)	36.7 (15.0)	37.5 (15.6)	36.3 (15.1)	1.000	1.000	0.992	1.000	0.04 (2.44)	0.985

Control = V1 Blank Label, V2 Blank Label; Experimental = V1 Blank Label, V2 Graphic Warning Label, V3 Graphic Warning Label.

A Holm’s method used to correct for multiple testing.

B Experimental – Control difference in change between visits 1 and 2, adjusting for race (NH white, NH black, other), age, sex, years of education, use of cigarettes or e-cigarettes (ever/never), and hookah use (last 30 days, lifetime use at private residence, and lifetime use at café; each coded as five times or less vs. more than 5 times). A negative value means a smaller increase in rating from visit 1 to visit 2 for the experimental group. Values were estimated using a linear mixed model.

C n = 44;

D n = 38.

E Bolded value indicates statistical significance, p<0.05.

**Table 3:** Direct Effects of Tobacco Collected Post Smoking Session - Measures are Mean (Standard Deviation).

						Intra-Group Comparisons: p-value^A^				Inter-Group Comparison of V1: V2 Change	
Measure	Control (C)		Experimental (E)			C	E			Adj Diff^B^	p-value
	V1	V2	V1	V2	V3	V1:V2	V1:V2	V2:V3	V1:V3		
No. Observ.	46	41	46	39	39						
Satisfying	49.8 (28.6)	49.5 (27.3)	48.9 (25.2)	52.3 (29.8)	44.7 (27.7)	1.000	1.000	0.075	0.444	−0.38 (5.72)	0.948
Pleasant	51.7 (29.8)	52.1 (29.0)	54.0 (26.6)	55.2 (30.2)	46.9 (29.5)	1.000	1.000	0.111	0.150	−3.43 (6.52)	0.600
Good Taste	57.7 (29.0)	49.8 (30.0)	53.9 (27.4)	48.6 (30.7)	44.0 (31.5)	0.052	0.331	0.331	**0.027** ^C^	−2.42 (6.07)	0.692
Bad Taste	17.7 (25.4)	14.8 (21.9)	15.0 (22.9)	14.5 (22.3)	10.8 (20.1)	0.876	0.876	0.508	0.577	4.92 (5.33)	0.359
Dizziness	17.3 (20.7)	14.7 (16.8)	15.2 (19.0)	12.6 (16.5)	13.0 (16.9)	1.000	1.000	1.000	1.000	2.38 (4.13)	0.567
Calmness	35.8 (31.0)	32.4 (28.3)	37.0 (30.5)	28.1 (26.6)	21.0 (26.3)	0.342	0.082	0.082	**0.007**	−10.01 (5.42)	0.069
Confusion	4.0 (8.9)	2.6 (4.1)	2.1 (3.5)	2.2 (3.8)	2.1 (3.7)	0.785	1.000	1.000	1.000	1.46 (1.47)	0.323
HelpedConcentration	16.8 (22.9)	12.5 (15.7)	10.3 (15.4)	11.1 (16.9)	10.8 (18.2)	0.701	1.000	1.000	1.000	1.35 (4.65)	0.772
More Awake	15.7 (19.6)	15.8 (23.4)	14.8 (22.7)	7.9 (16.1)	10.7 (18.0)	0.788	0.125	0.299	0.441	−9.72 (5.36)	0.074
Reduced Hunger	22.0 (30.1)	12.1 (18.7)	12.6 (18.9)	12.0 (17.2)	11.9 (18.3)	0.092	1.000	1.000	1.000	8.76 (6.12)	0.156
Sick	6.2 (11.1)	3.9 (6.8)	3.4 (7.0)	4.8 (10.4)	4.3 (10.1)	0.552	1.000	1.000	1.000	4.04 (2.65)	0.132
Sleepiness	17.6 (21.6)	11.4 (16.7)	17.4 (25.6)	13.3 (19.4)	13.3 (22.3)	0.454	1.000	1.000	1.000	2.31 (5.91)	0.697
Desire for Hookah Now	14.6 (21.3)	9.3 (12.0)	19.2 (28.0)	12.4 (23.2)	10.8 (21.0)	0.303	0.303	0.385	0.060	−0.67 (4.76)	0.889

Control = V1 Blank Label, V2 Blank Label; Experimental = V1 Blank Label, V2 Graphic Warning Label, V3 Graphic Warning Label.

A Holm’s method used to correct for multiple testing.

B Experimental – Control difference in change between visits 1 and 2, adjusting for race (NH white, NH black, other), age, sex, years of education, use of cigarettes or e-cigarettes (ever/never), and hookah use (last 30 days, lifetime use at private residence, and lifetime use at café; each coded as five times or less vs. more than 5 times). A negative value means a smaller increase in rating from visit 1 to visit 2 for the experimental group. Values were estimated using a linear mixed model.

C Bolded value indicates statistical significance, p < 0.05.

**Table 4:** Waterpipe-Modified Minnesota Withdrawal Data Collected Pre- and Post-Smoking - Measures are Mean (Standard Deviation).

						Intra-Group Comparisons: p-value^A^				Inter-Group Comparison of V1:V2 Change	
Measure	Control (C)		Experimental (E)			C	E			Adj Diff^B^	p-value
	V1	V2	V1	V2	V3	V1:V2	V1:V2	V2:V3	V1:V3		
No. Observ.	46	41	46	39	39						
Urge	−21.5 (26.0)	−18.8 (24.5)	−17.8 (27.5)	−14.3 (25.7)	−9.7 (21.1)	0.825	0.825	0.825	0.278	6.06 (6.43)	0.349
Anger	−1.5 (12.1)	−4.8 (16.3)	−3.8 (15.2)	−4.0 (18.4)	−1.8 (6.7)	0.567	0.981	0.901	0.891	4.89 (4.09)	0.235
Anxious	−2.9 (13.6)	−4.9 (14.7)	−0.7 (12.6)	−0.2 (8.6)	−2.9 (12.1)	1.000	1.000	0.618	1.000	1.09 (3.94)	0.783
Difficulty Concentrating	−1.1 (14.4)	−0.6 (8.5)	−1.6 (10.5)	−2.2 (11.2)	−1.2 (9.4)	1.000	1.000	1.000	1.000	0.76 (2.94)	0.796
Restlessness	−1.4 (11.1)	−0.3 (5.4)	−2.9 (15.9)	1.0 (4.2)	2.7 (11.5)	0.878	0.455	0.878	0.296	5.98 (3.20)	0.065
Hunger	1.0 (21.0)	0.9 (14.3)	8.6 (27.8)	0.4 (20.8)	4.3 (26.8)	1.000	0.417	1.000	1.000	−6.55 (5.52)	0.239
Impatient	−5.0 (16.6)	−4.0 (17.0)	−8.6 (22.8)	−4.2 (13.7)	−3.3 (18.4)	0.983	0.610	0.983	0.189	7.21 (3.66)	0.052
Craving Nicotine	−17.5 (26.9)	−14.0 (24.4)	−12.4 (26.7)	−9.8 (25.7)	−11.4 (24.5)	1.000	1.000	1.000	1.000	2.39 (6.25)	0.703
Drowsiness	5.8 (22.7)	0.1 (17.5)	7.5 (24.3)	0.7 (17.1)	−0.1 (16.3)	0.279	0.279	0.981	0.279	0.51 (5.59)	0.928
Depressed	−0.8 (9.5)	−1.5 (6.1)	−1.2 (7.4)	−1.4 (6.9)	−1.2 (8.2)	1.000	1.000	1.000	1.000	0.73 (2.36)	0.758
Sweets Desire	−0.2 (16.5)	−1.1 (11.9)	−0.8 (22.9)	−0.1 (7.4)	2.9 (22.5)	1.000	1.000	1.000	1.000	2.26 (5.43)	0.678

Control = V1 Blank Label, V2 Blank Label; Experimental = V1 Blank Label, V2 Graphic Warning Label, V3 Graphic Warning Label.

A Holm’s method used to correct for multiple testing.

B Experimental – Control difference in change between visits 1 and 2, adjusting for race (NH white, NH black, other), age, sex, years of education, use of cigarettes or e-cigarettes (ever/never), and hookah use (last 30 days, lifetime use at private residence, and lifetime use at café; each coded as five times or less vs. more than 5 times). A negative value means a smaller increase in rating from visit 1 to visit 2 for the experimental group. Values were estimated using a linear mixed model.

## Data Availability

Data will be made available on request.

## Appendix A. Supplementary data

Supplementary data to this article can be found online at https://doi.org/10.1016/j.addbeh.2025.108520.
