# Supplementary material for: Effect of graphic warning label on acute changes in waterpipe tobacco smoking behavior, biomarkers of exposure and harm, and subjective effects in a randomized trial
Source: Addict Behav. Author manuscript; Available in PMC 2026 Jun 15. (PMC13266813; doi:10.1016/j.addbeh.2025.108520)
Supplement: 1 [file NIHMS2184613-supplement-1.docx]

**SupplementaL Information**

**Effect of graphic warning label on acute changes in waterpipe tobacco smoking behavior, biomarkers of exposure and harm, and subjective effects in a randomized trial**

Marielle C. Brinkman,^1,2^ Toral Mehta,^2^ Michael L. Pennell,^1^ David Angeles,^1^ Soliana Kahassai,^1^ Chieh-Ming Wu,^1,2^ Hayley Curran,^2^ Brittney Keller-Hamilton,^2^ Elizabeth G. Klein,^1^ Megan E. Roberts,^1^ Paul Nini,^3^ Olorunfemi Adetona,^1^ Joanne G. Patterson,^1^ Darren Mays,^1^ Lucia Mandarano,^1^ Emma Jankowski,^1^ Annabelle Thomas,^1^ Amy K. Ferketich^1^

^1^ The Ohio State University, College of Public Health, Columbus, Ohio, USA

^2^ The Ohio State University Comprehensive Cancer Center, Center for Tobacco Research, Columbus, Ohio, USA

^3^ The Ohio State University, College of Arts and Sciences, Department of Design, Columbus, Ohio, USA

Corresponding Author:

Marielle C. Brinkman

324 Cunz Hall

1841 Neil Avenue

Columbus, OH 43210

[Brinkman.224@osu.edu](mailto:Brinkman.224@osu.edu)

**Supplemental Methods**

Waterpipe Configuration and Smoking Topography Data Collection

Briefly, for each smoking session, the RWP was fitted with a new hose (1.27 cm o.d., 0.84 cm i.d., Tygon Formula 2375, Saint-Gobain) and mouthpiece (7 cm long, 9.5 mm o.d., 7.8 mm i.d., Teflon®), and the blank or GWL was attached to the hose just below (1-2 cm below) the mouthpiece, as shown in Figure 1. The RWP head was filled with 15.0 ± 0.2 g tobacco in the participant’s preferred flavor (Double Apple, Mint, Gum Mint, Lemon Mint, Orange, and Rose flavors; all Nakhla, Egypt). The tobacco flavor each participant selected to smoke at V1 was also smoked for the remaining sessions. The head was covered with foil (Reynolds 625, 23x23 cm sheet, perforated with 37 holes). Participants were limited to one piece of quick-light charcoal (40 mm diameter, Three Kings, Holland), which was placed by study staff on top of the foil after it was ignited on an electric hot plate (Thermo Scientific Cimarec Analog Hot Plate 4x4, highest setting) for 100 s.37 Puffing flow rate data were continuously acquired (10 Hz) using a data acquisition card and USB chassis (NI-9205 and NI-cDAQ 9172, respectively, National Instruments, Austin, TX) and software (LabView 2017, National Instruments, Austin, TX) throughout the smoking session and later analyzed using an automated macro (MatLab R2023a, Mathworks, Natick, MA) to produce puffing topography measures. Sample weights of the tobacco and charcoal in/on the head were obtained using a top-loading balance (0.00 g precision) before and after smoking to calculate tobacco and charcoal consumption.

WP Smoking Protocol

Study personnel prepared the RWP and participants smoked *ad lib* to satiation, for a maximum of 60 minutes (no minimum smoking time was required). Participants were limited to one piece of quick-light charcoal and one head (15 g) of tobacco. Puffing topography was measured continuously throughout the session by the RWP. Exhaled CO was measured using a handheld electrochemical cell (CoVita Smokerlyzer, Santa Barbara, CA) before the smoking session and after 45 minutes of *ad lib* smoking (if the participant chose to smoke that long). If, after smoking for 45 minutes, a participant’s exhaled breath CO was greater than 50 ppm, the smoking session was discontinued and a final exhaled breath CO was collected. If not, the participant was allowed to continue smoking as they desired, for a maximum of 60 total minutes. Immediately following the smoking session, a final exhaled CO level was measured.

Measures

The Direct Effects of Nicotine Scale is a 10-item scale with responses on a 0-100 visual analogue scale. Questions ask about feelings “right now” and include nauseous, dizzy, lightheaded, nervous, among others. The Direct Effects of Tobacco Scale is a 13-item scale that asks participants to rate how they are feeling about hookah “right now.” Questions include, among others, “Was the hookah satisfying?” “Was the hookah pleasant?” and “Did the hookah taste good?”. Items are rated on a 0-100 visual analogue scale. The WP-modified Minnesota Withdrawal Sale is an 11-item scale that asks participants to rate how they are feeling “right now” on a 0-100 visual analogue scale. Feelings include urges to smoke, irritability/frustration/anger, anxious, among others. The Brief WP Questionnaire of Smoking Urges is a 10-item scale that asks questions about desire to smoke, anticipation of positive outcomes from smoking, anticipation of relief from nicotine withdrawal or from withdrawal-associated negative affect, and intention to smoke. Likert-type response options are on a 7-point scale from strongly disagree to strongly agree. Harm perceptions were assessed by asking, “How much do you think people harm themselves when they smoke hookah?” The response scale ranged from 0 (no harm) to 10 (extreme harm).

To characterize the sample, we examined the following: 1) demographic items (age, gender identity, race and ethnicity); 2) socioeconomic status growing up (“How would you best describe your family’s economic situation as you were growing up?” with options “We had barely enough to get by,” “We had enough to get by—but no more,” “We were solidly middle class,” “We had plenty of extras,” and “We had plenty of luxuries.”) and currently (“How would you describe your current social class?” with options “Working class/lower-middle class,” “Middle class,” “Upper-middle class,” “Upper class,” and “Other”; 3) years of formal education; 4) tobacco product use history; 5) WP use patterns; and 6) tobacco dependence (Lebanese Waterpipe Dependence Scale and the Hooked on Nicotine Checklist).

Randomization Procedure

A list of 292 unique sequential participant identification codes (IDs) was created and divided into 12 blocks (11 containing 8 IDs, one containing 4 IDs) based on numerical order. Within each block, half the IDs were randomly assigned to the Experimental (graphic warning label) group and the other half were assigned to the Control (Blank Label) group to ensure that each study arm was similar with respect to enrollment time of participants.

Data Analysis

Outcome data were analyzed using linear mixed models to account for incomplete, repeated measurements. All models contained fixed effects of visit, treatment group (Control, Experimental), condition-by-visit, and any baseline smoking or demographic factors that differed by group or were associated with dropout. Models for topography data on individual puffs (duration, flow, and volume) contained random subject and subject-by-visit interaction terms to account for the three-level hierarchy in the data (puffs within visit, visits within subject). For all other outcomes, we accounted for within subject correlations using an unstructured covariance matrix for the residual errors. Of primary interest was the difference in the change in outcomes from V1 to V2 by group, which was determined by the condition-by-visit interactions in our models. Intra-group comparisons of the changes over time were performed by stratifying our mixed models by group and performing multiple comparisons of the means at each visit. Holm’s method^[[1]](#footnote-1)^ was used to control overall type-I error rate across these comparisons. All analyses were performed using SAS Version 9.4 (SAS Inc, Cary, NC).

Sample size calculation

The sample size calculation for this study was based on the primary outcome of interest (change in total puff volume between weeks 1 and 2), primary comparison of interest (text/graphic vs. blank label) and our original plan to analyze the data using an ANCOVA model. The following formula from Oakes and Feldman^[[2]](#endnote-1)^ was used to calculate *n*, the number of participants per treatment arm:

$n=\frac{2{\sigma^{2}\left( 1-r^{2} \right)\left( Z_{\alpha/2}+Z_{\beta} \right)}^{2}}{\Delta^{2}}$,

where $\sigma^{2}$ is variance of total puff volume across smokers, *r* is the correlation between a smoker’s week 1 and week 2 total puff volume, Δ is the expected difference in change in puff volume between the optimized and no message arms, and $Z_{\alpha/2}$and $Z_{\beta}$correspond to the standard normal critical values for a two-sided type-I error rate of α and type-II error rate of β, respectively. Based on a previous study,^[[3]](#endnote-2)^ we expected a mean total puff volume of 42.2 L in each arm at Week 1, a variance of $\sigma^{2}$ = 387.4, and a within smoker correlation of *r* = 0.52. Assuming a two-sided type-I error rate of 0.05 and Δ= -10.6 L (chosen under assumption that smokers in the text/graphic label group would experience a 25% reduction in total puff volume and that there would no change in the blank label arm), a sample size of 39 participants per treatment arm would provide over 80% power. To account for possible loss-to-follow-up, we inflated our sample size by 10% and thus recruited a total of 44 participants per treatment arm (total N=88 participants). We planned on using multiple imputation to account for dropout. However, after study completion, we changed our analysis to a linear mixed model since our subsequent research demonstrated that the linear mixed model is a more powerful intent-to-treat analysis strategy than an ANCOVA applied to multiple imputed data sets.^[[4]](#endnote-3)^

**Table ST-1. Smoking Outcome Data: Dropouts Excluded.**

|  | | | | | | | **Intra-Group Comparisons: p-value^A^** | | | | **Inter-Group Comparison of V1:V2 Change** | |
| --- | --- | --- | --- | --- | --- | --- | --- | --- | --- | --- | --- | --- |
| **Measure** | **Units** | **Control (C)** | | **Experimental (E)** | | | **C** | **E** | | |  |  |
|  |  | **V1** | **V2** | **V1** | **V2** | **V3** | **V1:V2** | **V1:V2** | **V2:V3** | **V1:V3** | **Adj Diff^B^** | **p-value** |
| No. Observ. |  | 41 | 41 | 39 | 39 | 39 |  |  |  |  |  |  |
| **Consumption** | | | | | | | | | | | | |
| Tobacco Cons | g | 2.69 (1.35) | 2.83 (1.54) | 2.75 (1.29) | 2.63 (1.04) | 2.57 (1.12) | 1.000 | 1.000 | 1.000 | 1.000 | -0.36  (0.37) | 0.338 |
| Charcoal Cons | % | 68.7 (10.5)^C^ | 69.8 (10.8) | 68.1 (11.9)^D^ | 68.8 (11.8)^E^ | 66.6 (11.7) | 0.820 | 0.820 | 0.195 | 0.579 | 0.57  (2.42) | 0.814 |
| **Biomarker of Combustion** | | | | | | | | | | | | |
| CO Boost | ppm | 46.6 (28.5) | 46.5 (33.4) | 55.6 (36.8) | 47.6 (23.6) | 47.4 (25.3) | 1.000 | 0.291 | 1.000 | 0.439 | -4.5  (6.6) | 0.497 |
| **Puffing Topography** | | | | | | | | | | | | |
| Puff Volume | L | 0.97 (0.93) | 0.70 (0.46) | 1.15 (1.01) | 1.02 (0.91) | 0.78 (0.63) | 0.363 | 0.363 | 0.363 | 0.138 | -0.09 (0.21) | 0.684 |
| Puff Duration | s | 2.9 (1.7) | 2.6 (1.4) | 3.4 (1.9) | 3.1 (1.7) | 2.8 (1.4) | 0.568 | 0.568 | 0.273 | 0.076 | -0.1 (0.3) | 0.850 |
| Avg Puff Flow | L/min | 17.8 (8.1) | 16.0 (6.9) | 17.8 (8.0) | 17.5 (7.5) | 15.5 (6.4) | 0.685 | 0.841 | 0.841 | 0.685 | -0.7 (1.6) | 0.667 |
| Total Puff Vol | L | 63.9 (93.1) | 51.0 (46.4) | 63.7 (72.3) | 61.6 (74.5) | 44.8 (38.7) | 0.630 | 0.820 | 0.429 | 0.323 | -1.0 (17.4) | 0.952 |
| Total Puffs |  | 66.0 (58.0) | 72.5 (56.0) | 55.4 (38.0) | 60.6 (39.5) | 57.2 (38.3) | 0.505 | 0.358 | 0.759 | 0.759 | -1.3 (6.3) | 0.834 |
| Total Puffing Time | min | 3.2 (2.9) | 3.1 (2.2) | 3.2 (2.6) | 3.2 (2.5) | 2.7 (1.8) | 1.000 | 1.000 | 0.540 | 0.540 | -0.1 (0.4) | 0.826 |
| Total Smoke Time | min | 38.8 (14.8) | 39.0 (15.2) | 36.9 (15.9) | 37.5 (15.6) | 36.3 (15.1) | 1.000 | 1.000 | 0.988 | 1.000 | 0.1 (2.5) | 0.971 |

^A^ Holm’s method used to correct for multiple testing.

^B^ Experimental – Control difference in change between visits 1 and 2, adjusting for race (NH white, NH black, other), age, sex, years of education, use of cigarettes or e-cigarettes (ever/never), and hookah use (last 30 days, lifetime use at private residence, and lifetime use at café; each coded as five times or less vs. more than 5 times). A negative value means a smaller increase in from visit 1 to visit 2 for the experimental group. Values were estimated using a linear mixed model.

^C^ n = 40; ^D^ n = 37; ^E^ n = 38

Control = V1 Blank Label, V2 Blank Label; Experimental = V1 Blank Label, V2 Graphic Warning Label, V3 Graphic Warning Label

**Table ST-2. Direct Effects of Nicotine, Post-Pre-Smoking, Values Shown are Change from Baseline Visit (V1): Analysis Using All Available Data.**

|  | | | | | | | **Intra-Group Comparisons:**  **p-value^A^** | | | | **Inter-Group Comparison of V1:V2 Change** | |
| --- | --- | --- | --- | --- | --- | --- | --- | --- | --- | --- | --- | --- |
| **Measure** | **Units** | **Control (C)** | | **Experimental (E)** | | | **C** | **E** | | | **Adj Diff^B^** | **p-value** |
|  |  | **V1**  **Blank** | **V2**  **Blank** | **V1**  **Blank** | **V2**  **GWL** | **V3**  **GWL** | **V1:V2** | **V1:V2** | **V2:V3** | **V1:V3** |  |  |
| No. Observ. |  | 46 | 41 | 46 | 39 | 39 |  |  |  |  |  |  |
| Nauseous |  | 3.8 (11.5) | 2.3 (4.8) | 1.8 (11.9) | 1.5 (9.7) | 2.3 (10.0) | 1.000 | 1.000 | 1.000 | 1.000 | 3.907 (2.709) | 0.153 |
| Dizzy |  | 8.0 (16.8) | 6.3 (10.7) | 4.4 (14.1) | 3.7 (10.8) | 2.9 (6.4) | 1.000 | 1.000 | 1.000 | 1.000 | 2.777 (3.367) | 0.412 |
| Lightheaded |  | 15.8 (19.6) | 9.0 (12.3) | 11.3 (22.5) | 7.7 (14.7) | 6.8 (12.5) | 0.130 | 0.350 | 0.570 | 0.307 | 5.675 (4.476) | 0.209 |
| Nervous |  | -4.1 (16.9) | -1.2 (5.1) | 0.2 (3.7) | 1.5 (9.6) | 0.3 (1.0) | 0.814 | 1.000 | 1.000 | 1.000 | -0.927 (3.135) | 0.768 |
| Sweaty |  | 2.1 (6.1) | -0.1 (4.4) | -0.1 (2.9) | 0.5 (4.0) | 0.6 (7.8) | 0.190 | 1.000 | 1.000 | 1.000 | 2.502 (1.462) | 0.091 |
| Headache |  | 2.4 (12.1) | 1.6 (6.3) | 2.5 (9.2) | 0.2 (12.3) | 1.8 (6.1) | 1.000 | 1.000 | 1.000 | 1.000 | -1.424 (2.768) | 0.609 |
| Excessive Salivation |  | 1.7 (7.6) | -0.2 (8.1) | 2.5 (9.6) | 0.9 (15.6) | 0.6 (11.1) | 1.000 | 1.000 | 1.000 | 1.000 | 0.078 (3.454) | 0.982 |
| Heart Pounding |  | 3.3 (8.8) | 1.9 (6.5) | 2.7 (8.0) | 3.7 (12.8) | 2.5 (7.0) | 1.000 | 1.000 | 1.000 | 1.000 | 2.738 (2.690) | 0.312 |
| Confused |  | 1.5 (10.5) | 0.5 (1.5) | 0.0 (1.5) | 0.2 (1.7) | 0.8 (2.4) | 0.435 | 0.547 | 0.235 | **0.042^C^** | -1.473 (1.715) | 0.393 |
| Weak |  | 3.2 (11.5) | 1.7 (4.1) | 0.7 (4.9) | 1.2 (5.4) | 2.3 (5.6) | 0.831 | 0.831 | 0.831 | 0.556 | 3.119 (1.790) | 0.085 |
| Overall WP Harm Perception |  | 0.3 (1.1) | 0.0 (0.7) | 0.2 (0.9) | 0.4 (1.0) | 0.4 (0.8) | 0.791 | 1.000 | 1.000 | 1.000 | 0.558 (0.300) | 0.066 |

^A^ Holm’s method used to correct for multiple testing.

^B^ Experimental – Control difference in change between visits 1 and 2, adjusting for race (NH white, NH black, other), age, sex, years of education, use of cigarettes or e-cigarettes (ever/never), and hookah use (last 30 days, lifetime use at private residence, and lifetime use at café; each coded as five times or less vs. more than 5 times). A negative value means a smaller increase in rating from visit 1 to visit 2 for the experimental group. Values were estimated using a linear mixed model.

^C^ Bolded value indicates statistical significance, p<0.05.

Control = V1 Blank Label, V2 Blank Label; Experimental = V1 Blank Label, V2 Graphic Warning Label, V3 Graphic Warning Label

**Table ST-3. Questionnaire on Smoking Urges Data collected Pre- and Post-Smoking** **Values Shown are Change from Baseline Visit (V1).**

|  | | | | | | | **Intra-Group Comparisons:**  **p-value^A^** | | | | **Inter-Group Comparison of V1:V2 Change** | |
| --- | --- | --- | --- | --- | --- | --- | --- | --- | --- | --- | --- | --- |
| **Measure** | **Units** | **Control (C)** | | **Experimental (E)** | | | **C** | **E** | | | **Adj Diff^B^** | **p-value** |
|  |  | **V1** | **V2** | **V1** | **V2** | **V3** | **V1:V2** | **V1:V2** | **V2:V3** | **V1:V3** |  |  |
| No. Observ. |  | 46 | 41 | 46 | 39 | 39 |  |  |  |  |  |  |
| QSU Sum |  | -11.8 (7.5) | -13.5 (10.5) | -12.4 (9.2) | -12.1 (8.6) | -12.2 (9.3) | 0.372 | 1.000 | 1.000 | 1.000 | 1.157  (2.002) | 0.565 |
| Desire |  | -1.7  (1.3) | -1.6  (1.5) | -1.5  (1.8) | -1.5  (1.8) | -1.6  (1.4) | 1.000 | 1.000 | 1.000 | 1.000 | -0.084  (0.372) | 0.822 |
| Nothing Better |  | -0.9  (1.2) | -1.1  (1.4) | -0.9  (1.2) | -0.8  (1.1) | -0.8  (1.1) | 0.849 | 1.000 | 1.000 | 1.000 | 0.206  (0.311) | 0.509 |
| If Possible |  | -1.9  (1.7) | -1.8  (1.6) | -2.0  (2.0) | -2.0  (1.6) | -1.8  (1.7) | 1.000 | 1.000 | 1.000 | 1.000 | -0.023  (0.452) | 0.960 |
| More Control |  | -0.1  (0.8) | -0.8  (1.3) | -0.6  (1.2) | -0.6  (1.0) | -0.5  (1.0) | **0.016^C^** | 1.000 | 1.000 | 1.000 | 0.450  (0.325) | 0.171 |
| Want Right Now |  | -0.5  (1.0) | -0.9  (1.3) | -0.7  (1.1) | -0.7  (1.1) | -0.8  (1.3) | 0.172 | 1.000 | 1.000 | 1.000 | 0.123  (0.318) | 0.701 |
| Urge |  | -1.2  (1.6) | -1.4  (1.6) | -1.2  (1.6) | -1.1  (1.5) | -1.1  (1.7) | 1.000 | 1.000 | 1.000 | 1.000 | 0.249  (0.377) | 0.511 |
| Taste |  | -1.8  (1.4) | -1.6  (1.6) | -1.9  (1.5) | -1.6  (1.5) | -1.6  (1.6) | 1.000 | 1.000 | 1.000 | 1.000 | 0.104  (0.372) | 0.781 |
| Anything for Hookah |  | -0.1  (0.6) | -0.5  (0.9) | -0.2  (0.6) | -0.3  (0.7) | -0.5  (0.9) | **0.021** | 0.455 | 0.368 | 0.065 | 0.236  (0.218) | 0.285 |
| Less Depressed |  | -0.3  (1.0) | -0.5  (1.1) | -0.3  (1.0) | -0.6  (0.8) | -0.3  (0.7) | 0.422 | 0.145 | 0.102 | 0.825 | -0.319  (0.264) | 0.231 |
| Going to Smoke |  | -3.2  (1.5) | -3.3  (1.6) | -3.0  (1.8) | -3.0  (1.9) | -3.2  (1.9) | 1.000 | 1.000 | 1.000 | 1.000 | 0.172  (0.362) | 0.637 |

^A^ Holm’s method used to correct for multiple testing.

^B^ Experimental – Control difference in change between visits 1 and 2, adjusting for race (NH white, NH black, other), age, sex, years of education, use of cigarettes or e-cigarettes (ever/never), and hookah use (last 30 days, lifetime use at private residence, and lifetime use at café; each coded as five times or less vs. more than 5 times). A negative value means a smaller increase in from visit 1 to visit 2 for the experimental group. Values were estimated using a linear mixed model.

^C^ Bolded value indicates statistical significance, p<0.05.

Control = V1 Blank Label, V2 Blank Label; Experimental = V1 Blank Label, V2 Graphic Warning Label, V3 Graphic Warning Label

**Table ST-4. Changes in harm perceptions* by warning label group.**

| **Group** | **Control**  **(Blank Label)** | **Experimental**  **(Warning Label)** | **p-value** |
| --- | --- | --- | --- |
| Pre-V2 Absolute Harm Perception | 6.27 (2.16) | 6.18 (2.16) |  |
| Post-V2 Absolute Harm Perception | 6.29 (2.17) | 6.56 (2.49) |  |
| Difference | 0.02 (0.72) | 0.38 (1.02) | 0.07 |

*Participants were asked before and after each smoking session: *How much do you think people harm themselves when they smoke hookah*? Response options ranged from 0 (no harm) to 10 (extreme harm).

**Table ST-5. Association between Lebanese Water Pipe Dependency Scale (LWDS-11) and smoking behavior at Visit 1.**

| **Measure** | **N** | **Spearman Corr** | **95% Confidence Limits** | | **p-value** |
| --- | --- | --- | --- | --- | --- |
| Tobacco Cons (g) | 92 | 0.288 | 0.089 | 0.466 | 0.005 |
| Char Cons (%) | 88 | 0.133 | -0.079 | 0.333 | 0.216 |
| CO Boost (ppm) | 92 | 0.237 | 0.034 | 0.422 | 0.022 |
| Total Puff Vol (L) | 92 | 0.186 | -0.020 | 0.376 | 0.075 |
| Total Puffs | 92 | 0.216 | 0.012 | 0.403 | 0.037 |
| Total Puffing Time (min) | 92 | 0.193 | -0.012 | 0.383 | 0.064 |
| Smoking Duration (min) | 92 | 0.082 | -0.125 | 0.282 | 0.438 |

**Table ST-6. Differences in tobacco flavor by warning label group.**

| **Group** | **Double Apple** | **Mint** | **Orange/Rose** |
| --- | --- | --- | --- |
| Control (Blank Label) | 14 (30.4%) | 20 (43.5%) | 12 (26.1%) |
| Experimental (Warning Label) | 19 (41.3%) | 18 (39.1%) | 9 (19.6%) |

**Table ST-7. Differences in smoking behavior at visit 1 by tobacco flavor. Values listed under each flavor are mean (SD).**

| **Measure** | **Double Apple**  **(N = 33)** | **Mint**  **(N = 38)** | **Orange/Rose**  **(N = 21)** | **p-value^c^** |
| --- | --- | --- | --- | --- |
| Tobacco Cons (g) | 3.29 (0.87) | 2.37 (1.38) | 2.58 (1.45) | < 0.001 |
| Char Cons (%) | 69.3 (0.09) | 67.5 (11.9)^a^ | 68.5 (11.6)^b^ | 0.917 |
| CO Boost (ppm) | 47.7 (20.8) | 57.7 (40.6) | 49.5 (34.2) | 0.837 |
| Total Puff Vol (L) | 43.3 (50.3) | 73.3 (80.6) | 75.3 (105.3) | 0.397 |
| Total Puffs | 53.5 (34.1) | 68.0 (54.0) | 60.0 (53.8) | 0.708 |
| Total Puffing Time (min) | 2.61 (1.87) | 3.47 (2.72) | 3.52 (3.40) | 0.567 |
| Smoking Duration (min) | 39.0 (14.9) | 36.1 (14.6) | 37.4 (15.3) | 0.854 |

**^a^**N = 36, ^b^N = 19, ^c^From Kruskal-Wallis test.

REFERENCES

1. Holm A. A simple sequentially rejective multiple test procedure - pdf4R8xPVzCnX.pdf. Scandinavian Journal of Statistics. 1979;6(2):65. [↑](#footnote-ref-1)
2. Oakes JM, Feldman HA. Statistical power for nonequivalent pretest-posttest designs the impact of change-score versus ANCOVA. Evaluation Review 2001;25(1):3{28.78. Enders CK. Multiple imputation as a flexible tool for missing data handling in clinical research. . Beh Res Ther. 2016;7967(16):30195. [↑](#endnote-ref-1)
3. Kim H, Brinkman MC, Sharma E, Gordon SM, Clark PI. Variability in Puff Topography and Exhaled CO in Waterpipe Tobacc...: Ingenta Connect. Tobacco Regulatory Science. 2016;2(4):301. [↑](#endnote-ref-2)
4. Xi W, Pennell ML, Andridge RR, Paskett ED. Comparison of intent-to-treat analysis strategies for pre-post studies with loss to follow-up. Contemp Clin Trials Commun. 2018 May 9;11:20-29.  [↑](#endnote-ref-3)
